# Development of two short FFQ to assess diet quality in UK pre-school and primary school-aged children based on National Diet and Nutrition Survey data

**DOI:** 10.1017/S0007114525103449

**Published:** 2025-05-14

**Authors:** Morgan Mason, Sarah C. Shaw, Janis Baird, Millie Barrett, Donna Lovelock, Kathryn Woods-Townsend, Keith M. Godfrey, Christina A. Vogel, Sarah R. Crozier

**Affiliations:** 1 NIHR Southampton Biomedical Research Centre, University of Southampton and University Hospital Southampton NHS Foundation Trust, Southampton, UK; 2 School of Healthcare Enterprise and Innovation, Faculty of Medicine, University of Southampton, Southampton SO16 6YD, UK; 3 MRC Lifecourse Epidemiology Centre, University of Southampton, University Hospital Southampton, Southampton SO16 6YD, UK; 4 NIHR Applied Research Collaboration Wessex, Southampton Science Park, Innovation Centre, 2 Venture Road, Chilworth, Southampton SO16 7NP, UK; 5 Centre for Food Policy, City St George’s, University of London, Northampton Square, London EC1V 0HB, UK

**Keywords:** Dietary assessment, Diet quality, Principal component analysis, FFQ, Young children

## Abstract

Assessing children’s diets is currently challenging and burdensome. Abbreviated FFQ have the potential to assess dietary patterns in a rapid and standardised manner. Using nationally representative UK dietary intake and biomarker data, we developed an abbreviated FFQ to calculate dietary quality scores for pre-school and primary school-aged children. UK National Diet and Nutrition Survey (2008–2016) weekly consumption frequencies of 129 food groups from 4-d diaries were cross-sectionally analysed using principal component analysis. A 129-item score was derived, alongside a 12-item score based on foods with the six highest and six lowest coefficients. Participants included 1069 pre-schoolers and 2565 primary schoolchildren. The first principal component explained 3·4 and 3·0 % of the variation in the original diet variables for pre-school and primary school groups, respectively, and described a prudent diet pattern. Prudent diet scores were characterised by greater consumption of fruit, vegetables and tap water and lower consumption of crisps, manufactured coated chicken/turkey products, purchased chips and soft drinks for both age groups. Correlations between the 129-item and 12-item scores were 0·86 and 0·84 for pre-school and primary school-aged children, respectively. Bland–Altman mean differences between the scores were 0·00 sd; 95 % limits of agreement were −1·05 to 1·05 and −1·10 to 1·10 sd for pre-school and primary school-aged children, respectively. Correlations between dietary scores and nutritional biomarkers showed only minor attenuation for the 12-item compared with the 129-item scores, illustrating acceptable congruence between prudent diet scores. The two 12-item FFQ offer user-friendly tools to measure dietary quality among UK children.

Childhood overweight and obesity are linked to obesity in adolescence and adulthood^(1)^ and to increased risk of chronic diseases in later life^(2,3)^. Overweight and obesity can reduce children’s self-esteem and are associated with lower educational attainment and greater learning difficulties^(4–6)^. In 2022/2023 in England, 22·7 % of year 6 children were living with obesity. Historical trends show that obesity rates double between reception and year 6 from 10 to 23 %, respectively, over this 7-year period^(7,8)^. The burden of obesity is not spread equally among society; children living in the most deprived areas are more than twice as likely to have obesity as those from more affluent areas^(8)^. Of public health concern, the COVID-19 pandemic exacerbated this gap, indicating an urgent need to monitor and improve children’s dietary quality^(9,10)^.

Dietary intake is a complex behaviour, and analysis of dietary patterns is being increasingly recognised as critical to addressing rising rates of obesity and chronic diseases^(11–13)^. Dietary patterns embrace the interrelationships and synergies between foods and eating practices and have been shown to be more strongly associated with health outcomes than single nutrients or foods^(12)^. Techniques such as principal component analysis (PCA) can reveal discriminative dietary patterns, and the results of such analyses have been shown to be predictive of mortality, morbidity and disease-related biomarkers^(14,15)^. Dietary pattern recommendations associated with a healthful diet, classified as a ‘prudent’ dietary pattern, exhibit similar characteristics globally^(16)^. Prudent diets are characterised by minimal consumption of foods high in saturated fat, salt and sugar and higher intakes of fruit, vegetables, whole grain and water^(14)^.

Alternative dietary assessment methods, such as 24 h recall, multi-day food diaries and full FFQ^(17)^, for dietary patterns can hamper evaluations in large-scale research studies due to the high participant burden. These dietary assessment approaches can be further complicated when evaluating child populations where low cognitive abilities, lack of food knowledge, memory and understanding of frequency or portion sizes can contribute to misreporting of food and drink intake^(18,19)^. Previously, we developed an abbreviated 20-item FFQ for use among young women that showed a strong correlation with a larger 100-item FFQ in determining dietary quality. The short FFQ showed clear advantages in reduction of resources and ease of administration in epidemiological and intervention studies^(20–22)^. These advantages have been noted in similar diet quality studies outside of the UK^(18,23,24)^ where the use of abbreviated FFQ to ascertain dietary scores in children has been praised for similar reasons.

A systems-based childhood obesity intervention, Early LifeLab (ELL), is currently ongoing within primary schools in Southampton, UK. Modelled after the success of LifeLab^(25,26)^, ELL provides a multi-component intervention consisting of teacher continuing professional development and engaging, interactive science-based classroom modules centred around engaging children in the importance of healthy choices regarding the food they eat, their sleep hygiene and physical activity^(27)^. To evaluate the impact of the ELL intervention on diet, an FFQ is needed to assess dietary quality. This assessment measure must be easy for teachers to administer and not too burdensome for children to complete in the classroom. To the authors’ knowledge, no abbreviated FFQ exists to assess dietary quality that is nationally representative of the diets of pre-school and primary schoolchildren in the UK. The aim of this paper is to address this gap in evidence by developing two short FFQ that can be used to calculate dietary scores among pre-school (aged 1–3 years) and primary school (aged 4–11 years) children living in the UK. These measures were developed using data from the National Diet and Nutrition Survey (NDNS)^(27)^, and comparisons were made between the full and reduced-item scores alongside assessment of associations between both scores and nutritional biomarkers.

## Methods

### The National Diet and Nutrition Survey

Beginning in 2008, the NDNS is a continuous programme consisting of cross-sectional assessments of diet, nutrient intake and nutritional status across the general UK population. A nationally representative collection of data from 1000 (500 adults and 500 children) UK residents aged 1·5 years and older is undertaken each year^(28)^. A selection of households from all residential addresses in the UK is randomly selected from clusters of small geographical areas based on postcode. With an effort to maintain a balanced representation of adults and children, a random selection of both groups within each household is selected to participate in the survey^(29)^. The NDNS is conducted in adherence to the Declaration of Helsinki and the MRC Good Research Practice principles. Approval for all procedures was obtained from the Oxfordshire-A Research Ethics Committee, and informed consent was acquired from all participants. For the purposes of this analysis, data for 1069 pre-schoolers (aged 1–3 years) and 2565 primary school-aged participants (aged 4–11 years) from NDNS years 1–8 (2008–2016) were accessed through the UK Data Service archives^(30)^.

Face-to-face interviews at home were used to gather details concerning the health behaviours and sociodemographic traits of each participant and their household during the initial phase of the surveys. In addition, height and weight were also measured. Ethnicity was classified as white or all other ethnic groups combined. Data on household income were disclosed by the primary food provider for the household and normalised to accommodate variation in resource demands, such as household size and composition, to calculate an equivalised household income. The household address was used to determine the Index of Multiple Deprivation scores^(31)^.

Following the visit, a parent or carer was asked to complete a 4-d (unweighed) food diary, recording all food and drinks consumed both at home and away from home, along with estimated portion size, brand names or ingredients for homemade meals. The child was able to help where appropriate. The food diary was checked at a further follow-up visit. Data from food diaries were coded by trained coders from the NDNS research team into 155 groups of nutritionally similar food groups (NDNS subsidiary food groups). A total of 126 (11·8 %) of pre-schoolers and 584 (22·8 %) of primary school-aged children were willing to provide a blood sample, which was collected by a nurse at the second interview. A total of 973 (37·9 %) of primary school-aged children (and no pre-schoolers) were willing to provide a urine sample.

### Dietary data

Among the NDNS subsidiary food groups, ten groups relating to alcoholic drinks and sixteen groups relating to dietary supplements were excluded as being inappropriate for these age groups. Thus, 129 food groups were included in this study’s analyses.

Implementation of a previous short FFQ^(20)^ demonstrated groupings such as ‘other fruit’ are difficult to include in standalone FFQ. To avoid an ‘other fruit’ category in the final short FFQ, this NDNS subsidiary food group was disaggregated using NDNS food codes into six groups: ‘peaches, plums, cherries, grapes and blueberries’, ‘tropical fruits’, ‘cooked fruit’, ‘dried fruit’, ‘strawberries and raspberries’ and remaining ‘other fruit’. These groups were based on those in a previous FFQ that has been used widely in Southampton nutrition studies^(32)^. The six soft drinks categories were collapsed into one category to aid interpretation of the final results. Frequencies of consumption of the 129 food groups were derived from diary data and converted to weekly frequencies. For the 2·5 % of pre-schoolers and 1·7 % of primary school-aged children with 3-d diaries, the frequencies were multiplied by 4/3, respectively.

### Principal component analysis

PCA was used to identify dietary patterns. PCA is a statistical technique that produces new variables that are uncorrelated linear combinations of the original dietary variables and maximise the explained variance within the original variables^(33)^. PCA was performed on the reported weekly frequencies of consumption of the 129 foods of pre-schoolers and primary school-aged children separately. For both age groups, the first principal component was retained. Individual dietary pattern scores were calculated by multiplying the PCA coefficients for the 129 food groups by each individual participant’s standardised reported frequencies of consumption and then summing across all food groups to provide a score for every participant.

### Creating a reduced-item score

To create a reduced-item score for both age groups, foods with the six highest positive and six lowest negative coefficients from the PCA were chosen. The decision to choose these twelve foods, rather than those of the largest absolute coefficient values (highest magnitude regardless of positive or negative sign), was made to detect reductions in consumption of less healthy foods as well as increases in consumption of healthier foods. Both these aspects of diet have clinical relevance and are incorporated into national dietary guidelines, hence important to include^(14,15)^. For both age groups, a twelve-item score was calculated by multiplying the PCA coefficients for the twelve identified food groups by each individual participant’s standardised frequency of consumption and summing across the twelve food groups.

All four scores (the 129-item and 12-item pre-school scores and the 129-item and 12-item primary school-aged scores) had a right-hand skew. The following transformations were applied in order to create dietary scores with a normal distribution: after adding six to each variable (so all values were positive), scores were logged and then standardised by subtracting the mean and dividing by the standard deviation. All scores therefore have a mean of zero and a standard deviation of one.

### Biomarkers

Biomarkers were chosen that reflect diets with (i) high intakes of fruit and vegetables (beta-carotene, total carotenoids and serum folate), (ii) a desirable lipid profile (HDL-cholesterol and TAG) and (iii) low sodium intake (urinary sodium). These biomarkers are also of clinical relevance to cardiometabolic health^(34–39)^. UK folic acid fortification of flour was not mandated until 2021, after the data collection period of this study, and hence does not impact these analyses^(40)^. Carotenoids were measured by high-performance liquid chromatography^(41)^. Serum folate was measured by LC-MS/MS (in years 7 and 8 only) and urinary sodium using ion-specific electrodes on the Siemens Dimension® Xpand clinical chemistry system with the QuikLYTE® module. HDL-cholesterol was directly measured using an AHDL-cholesterol assay, and TAG were measured using lipoprotein lipase and glycerol kinase enzyme reagents, both on the Siemens Dimension® RXL in years 1–5 and the Dimension® Xpand in years 6–8. Further details on the laboratory and assay methods are available elsewhere^(29,42)^. TAG and urinary sodium were not measured in pre-schoolers.

### Statistical analysis

In descriptive analyses, non-normally distributed continuous variables were summarised using medians and interquartile ranges; for categorical variables, frequencies and percentages were used. Associations between the full 129-item dietary scores and the short 12-item dietary scores were calculated using Pearson’s correlation coefficient. Bland–Altman 95 % limits of agreement^(43)^ were also calculated to assess the level of agreement between the full 129-item and short 12-item scores. Spearman’s correlations were used to assess the associations between the dietary scores and biomarkers, with 95 % CI derived using bootstrapping with 1000 replications. Mean twelve-item dietary scores were calculated according to sociodemographic variables, and *t* tests were used to test for differences in dietary scores between binary groups (sex and ethnicity). Spearman’s correlations were used to compare dietary scores and continuous variables (BMI, Index of Multiple Deprivation and equivalised household income). Analyses were performed using Stata 14.1^(44)^.

### Public and patient involvement

Following statistical analyses, patient and public involvement (PPI) work was carried out to ensure face validity and accessibility of the abbreviated FFQ for primary school-aged children. Four public contributors participated in PPI activities. They were women aged 31–45 years, living across different regions of England (i.e. greater areas of Southampton and Manchester), identifying as being from differing ethnicities and being parents of primary school-aged children. One public contributor identified as having dyslexia/dyspraxia, and one contributor identified as having two children with complex learning difficulties. Feedback concerned the wording and design of the questionnaire. The terminology of the FFQ was revised accordingly. For example, adding the word ‘cordial’ alongside ‘squash’ and adding examples of specific foods for food categories (i.e. almonds and walnuts).

## Results

### Participant characteristics

NDNS data for 1069 pre-schoolers (aged 1–3 years) and 2565 primary school-aged participants (aged 4–11 years) are described in Table 1. Approximately half the sample were female (48 % pre-schoolers and 47 % primary school-aged). In both groups, a large proportion (86 %) were of white ethnicity, and 40 % lived in areas that were classified in the two least deprived fifths in the UK, demonstrating the representativeness of the NDNS sample in terms of deprivation.

**Table 1.** Characteristics of the 1069 pre-schoolers and 2565 primary school-aged children in the NDNS (Numbers and percentages; median values and interquartile ranges)

	Pre-schoolers		Primary school-aged	
Characteristic	*n*	%	*n*	%
Female	516	48 %	1210	47 %
	Median	IQR	Median	IQR
Age (years)	2·2	0·7	7·4	2·3
BMI (kg/m^2^)	16·5	15·5–17·6	16·6	15·5–18·4
	*n*	%	*n*	%
White ethnicity	922	86 %	2211	86 %
Two least deprived fifths IMD^*^	276	40 %	671	40 %
	Median	IQR	Median	IQR
Equivalised household income (£/year)	23 305	13 779–38 990	21 929	12 773–36 016

NDNS, National Diet and Nutrition Survey; IQR, interquartile range.

* Available for 698 pre-schoolers and 1674 primary school-aged children.

### Principal component analysis

The first component of the PCA explained 3·4 % of the variation in the original dietary variables among pre-schoolers and 3·0 % of the original variables among primary school-aged children (online Supplementary Table 1). Consistency between the magnitude of coefficients between the analyses in the two age groups is notable. High scores on these components were achieved by consuming rice, wholemeal bread, eggs and egg dishes, salad vegetables, beans and pulses, nuts and seeds, fruit, fruit juice and tap water frequently, as well as white bread, manufactured coated chicken and turkey products, purchased burgers and kebabs, manufactured meat pies and pastries, purchased chips and other manufactured potato products and soft drinks infrequently. This pattern described a diet consistent with UK dietary recommendations^(45,46)^. The resulting individual scores were termed ‘prudent’ diet scores and were consistent with previously published studies covering different stages of the lifecourse^(13,14,20)^. The coefficients used to create the 129-item prudent diet score for pre-school-aged children and primary school-aged child are presented in online Supplementary Table 1. The twelve most discriminatory foods in each age group and their corresponding coefficients are shown in Table 2.

**Table 2.** Coefficients for the twelve most discriminatory foods in pre-schoolers and primary school-aged children

Food	Pre-schoolers	Primary school-aged
Tap water only	0·24	0·24
Salad and other raw vegetables	0·23	0·26
Tomatoes (raw)	0·20	0·23
Carrots (raw)		0·20
Nuts and seeds		0·19
Tropical fruit		0·18
Peaches, plums, cherries, grapes and blueberries	0·20	
Fruit juice	0·18	
Apples and pears not canned	0·16	
Meat pies and pastries (manufactured)		–0·09
White bread (not high fibre or multiseed)		–0·10
Crisps and savoury snacks	–0·09	–0·11
Burgers and kebabs purchased	–0·09	
Other manufactured potato products fried/baked	–0·11	
Manufactured coated chicken/turkey products	–0·17	–0·15
Chips, purchased including takeaway	–0·18	–0·16
Soft drinks	–0·21	–0·19

Table 3 shows the median frequency of consumption of food groups according to quarters of the 129-item and short 12-item pre-schooler prudent diet scores. These summary statistics demonstrate notable increases in median frequency of consumption of fruit, vegetables, brown and wholemeal bread and water across quarters of both the full short scores. Similarly, decreases in median consumption of chips, manufactured meat products, white bread and soft drinks were observed across quarters. Very similar patterns of intakes of these food groups were seen across quarters of the prudent diet score among primary school-aged children (Table 4).

**Table 3.** Median frequency of consumption of selected food groups per week in quarters of the pre-schoolers’ prudent diet scores

Comparison with full 129-item pre-schooler prudent diet scores				
Food group	Least prudent diet	−0·7 to 0·00 sd	0·00 to 0·7 sd	Most prudent diet
	−3·9 to −0·7 sd			0·7 to 3·2 sd
Fruit	5·3	8·8	12·3	19·3
Vegetables	4·7	7·0	8·8	14·0
Brown and wholemeal bread	0	0	1·75	3·5
Water	0	3·5	8·8	17·5
Chips, not homemade	1·8	1·8	0	0
Manufactured meat products	1·8	1·8	0	0
White bread	5·3	5·3	3·5	3·5
Soft drinks	28·0	15·8	10·5	3·5

Comparison with short 12-item pre-schooler prudent diet scores				
Food group	Least prudent diet	−0·7 to 0·00 sd	0·00 to 0·7 sd	Most prudent diet
	−4·1 to −0·7 sd			0·7 to 3·2 sd
Fruit	7·0	10·5	12·3	19·3
Vegetables	5·3	7·0	8·8	14·0
Brown and wholemeal bread	0	0	1·8	3·5
Water	0	3·5	10·5	17·5
Chips, not homemade	1·8	1·8	0	0
Manufactured meat products	1·8	0	0	0
White bread	5·3	5·3	3·5	3·5
Soft drinks	31·5	17·5	8·8	1·8

**Table 4.** Median frequency of consumption of selected food groups per week in quarters of the primary school-aged prudent diet scores

Comparison with full 129-item primary school-aged prudent diet scores				
Food group	Least prudent diet	−0·7 to 0·00 sd	0·00 to 0·7 sd	Most prudent diet
	−5·8 to −0·7 sd			0·7 to 3·9 sd
Fruit	3·5	5·3	8·8	14·0
Vegetables	3·5	7·0	10·5	17·5
Brown and wholemeal bread	0	1·8	1·8	3·5
Water	3·5	5·3	8·8	14·0
Chips, not homemade	1·8	1·8	1·8	0
Manufactured meat products	1·8	1·8	0	0
White bread	7·0	5·3	5·3	3·5
Soft drinks	19·3	15·8	10·5	5·3

Comparison with short 12-item primary school-aged prudent diet scores				
Food group	Least prudent diet	–0·7 to 0·00 sd	0·00 to 0·7 sd	Most prudent diet
	–3·6 to −0·7 sd			0·7 to 3·9 sd
Fruit	3·5	7·0	8·8	12·3
Vegetables	3·5	7·0	9·3	17·5
Brown and wholemeal bread	0	1·8	1·8	3·5
Water	1·8	5·3	10·5	15·8
Chips, not homemade	3·5	1·8	1·8	0
Manufactured meat products	1·8	1·8	0	0
White bread	7·0	5·3	5·3	3·5
Soft drinks	24·5	14·0	8·8	3·5

**Figure 1.**
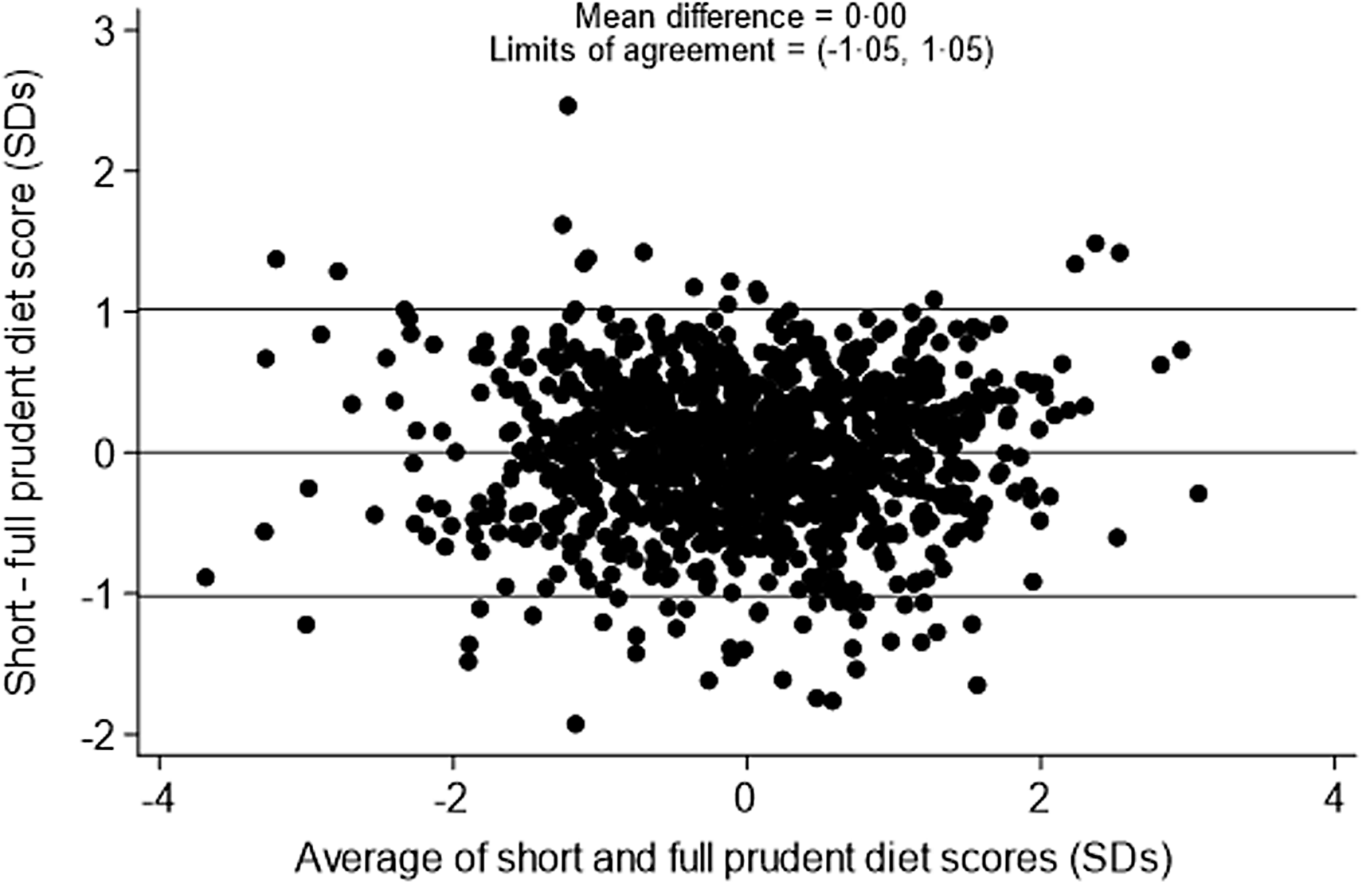
Bland–Altman plot to show agreement between the 129-item and 12-item prudent diet score among pre-schoolers.

**Figure 2.**
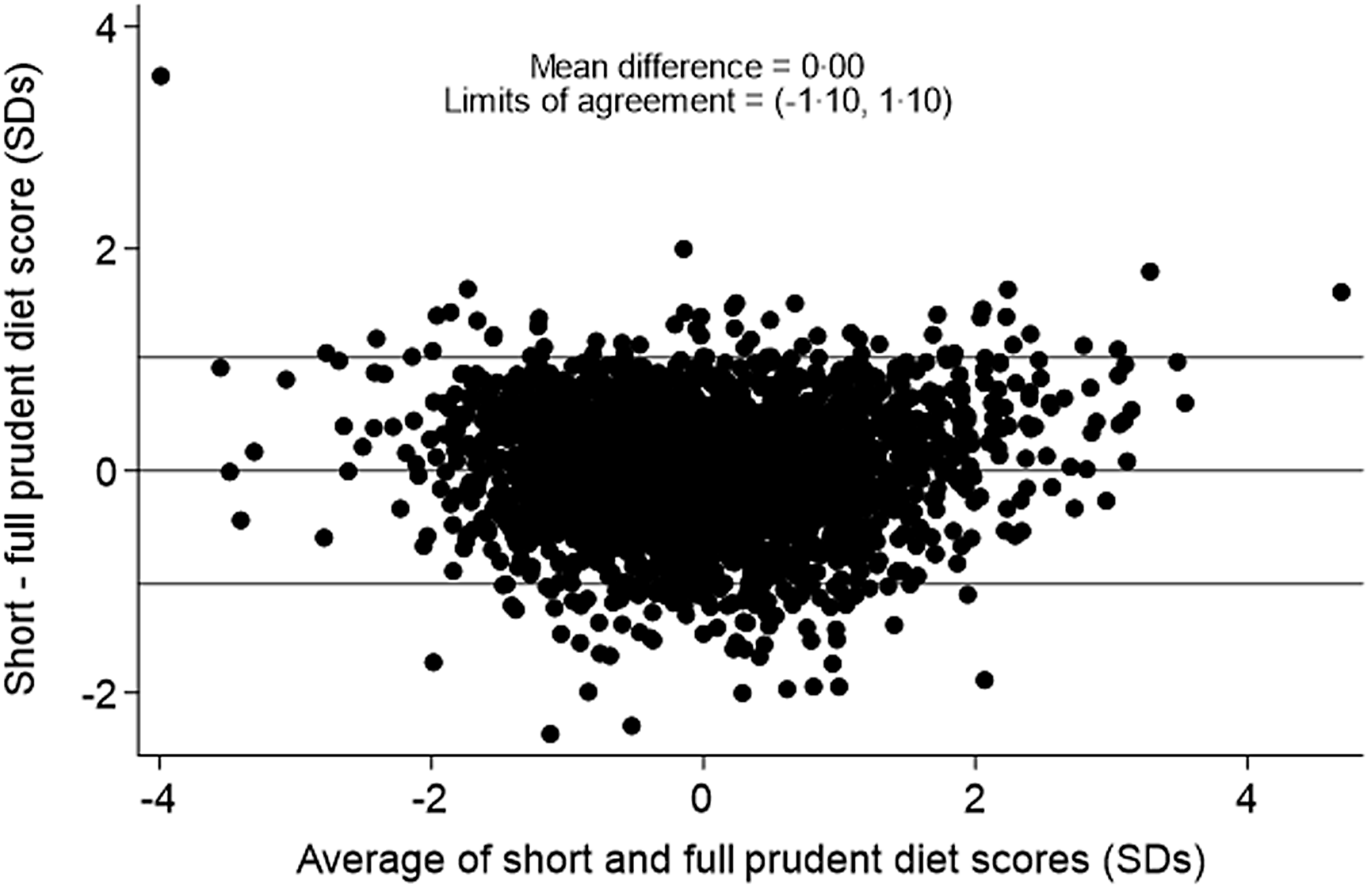
Bland–Altman plot to show agreement between the 129-item and 12-item prudent diet score among primary school-aged children.

The correlation between the full 129-item prudent diet score and the short 12-item prudent diet score was 0·86 among pre-schoolers and 0·84 among primary school-aged children. A Bland–Altman plot of the limits of agreement between the two scores among pre-schoolers (Fig. 1) showed a mean difference of 0·00 sd and 95 % limits of agreement of −1·05 to 1·05 sd. The Bland–Altman plot of the limits of agreement between the two scores among primary school-aged children (Fig. 2) showed a mean difference of 0·00 sd and 95 % limits of agreement of −1·10 to 1·10 sd.

### Associations with biomarkers

Biomarker data were available for a relatively small number of children (2–8 % of pre-schoolers and 5–38 % of primary school-aged children). Spearman correlation coefficients were generally in the expected directions for the full 129-item and short 12-item prudent diet scores (Table 5). Positive correlations were observed between the prudent diet scores and beta-carotene, total carotenoids and serum folate. Patterns were less consistent for HDL-cholesterol. Negative correlations were observed between TAG and urinary sodium and both primary school-aged children’s full and short prudent diet scores (data not available for pre-school age). The strongest correlation among pre-school-aged children was between the full 129-item prudent diet score and serum folate (*r*_s_ = 0·27, 95 % CI −0·15, 0·70, *P* = 0·19), and this association only slightly attenuated with the short 12-item prudent diet score (*r*_s_ = 0·26, 95 % CI −0·16, 0·28, *P* = 0·21). Similarly, the strongest correlation among primary school-aged children was with serum folate and this remained consistent for the short 12-item score (both *r*_s_ = 0·38, 95 % CI 0·024, 0·53 and 95 % CI 0·22, 0·53, respectively, both *P* < 0·001).

**Table 5.** Spearman correlation coefficients between prudent diet scores and biomarkers

Biomarker	Full 129-item prudent diet score r_s_	95 % CI	*P*	Short 12-item prudent diet score r_s_	95 % CI	*P*	*n*
Pre-schoolers							
Beta-carotene (µmol/l)	0·23	0·02, 0·44	0·04	0·19	–0·01, 0·40	0·08	83
Total carotenoids (µmol/l)	0·14	–0·07, 0·36	0·19	0·14	–0·07, 0·36	0·19	83
Serum folate (nmol/l)	0·27	–0·15, 0·70	0·19	0·26	–0·16, 0·28	0·21	25
HDL-cholesterol (mmol/l)	–0·06	–0·30, 0·17	0·58	0·04	–0·19, 0·27	0·72	77
Primary school-aged children							
Beta-carotene (µmol/l)	0·30	0·21, 0·39	< 0·001	0·26	0·17, 0·34	< 0·001	488
Total carotenoids (µmol/l)	0·32	0·24, 0·40	< 0·001	0·27	0·19, 0·35	< 0·001	488
Serum folate (nmol/l)	0·38	0·24, 0·53	< 0·001	0·38	0·22, 0·53	< 0·001	122
HDL-cholesterol (mmol/l)	0·09	–0·01, 0·19	0·07	0·07	–0·02, 0·17	0·14	402
TAG (nmol/l)	–0·11	–0·20, −0·02	0·01	–0·11	–0·20, −0·03	0·009	523
Urinary sodium (g/l)	–0·12	–0·19, −0·06	< 0·001	–0·09	–0·16, −0·03	0·004	973

*r*_s_, Spearman correlation coefficient, *P*-value, probability value.

### Sociodemographic characteristics

Associations between the twelve-item prudent diet scores and sociodemographic characteristics are shown in Table 6. No associations were apparent between the twelve-item prudent diet score and sex or BMI among pre-schoolers. Among primary school-aged children associations showed higher dietary quality scores for girls than for boys and better dietary quality among children with a lower BMI.

**Table 6.** Mean (SD) twelve-item prudent diet scores (SD) by sociodemographic characteristics (Numbers; mean values and standard deviations)

	Pre-schoolers				Primary school-aged			
Characteristic	*n*	Mean	sd	*P*	*n*	Mean	sd	*P*
Sex								
Male	553	0·02	1·04	0·42	1355	–0·06	1·00	0·002
Female	516	–0·03	0·95		1210	0·06	1·00	
BMI								
≤ 16·5 kg/m^2^	388	0·02	1·01	0·85^*^	1158	0·09	1·02	< 0·0001^*^
> 16·5 kg/m^2^	390	0·05	0·99		1296	–0·07	0·98	
Ethnicity								
White	922	–0·05	1·02	0·0002	2211	–0·07	1·00	< 0·0001
All other ethnic groups	147	0·28	0·85		354	0·42	0·91	
IMD								
Two least deprived fifths	276	0·33	1·00	< 0·0001^*^	671	0·22	1·03	< 0·0001^*^
Three most deprived fifths	422	–0·11	1·01		1003	–0·12	0·95	
Equivalised household income								
≤ £27 000	569	–0·23	0·99	< 0·0001^*^	1489	–0·15	0·97	< 0·0001^*^
> £27 000	418	0·31	0·94		874	0·26	1·01	

*P*-value, probability value; IMD, Index of Multiple Deprivation.

* *P*-value calculated using continuous characteristic data.

Prudent diet scores were notably lower among white children compared with children from all other ethnic groups combined, across both age groups, indicating poorer dietary quality among white children. Prudent diet scores were higher among more socio-economically advantaged participants. Better dietary quality is illustrated by the markedly higher mean scores among both age groups among children from more affluent neighbourhoods and living in households with higher equivalised income.

## Discussion

### Statement of principal findings

This study demonstrates the ability to assess dietary quality in UK pre-school and primary school-aged children through FFQ covering consumption of only twelve food groups. Principal component analysis (PCA) was applied to a nationally representative dataset, the NDNS, resulting in two sets of 129 coefficients representative of the dietary quality of young and very young children in the UK. A prudent diet pattern was identified through the PCA results, and a dietary quality score was calculated for each individual participant. Higher overall scores aligned with a diet which closely aligned with the UK dietary guidelines, including greater consumption of fruits and vegetables with lower consumption of refined sugar, processed meats and higher saturated fats^(45)^. Dietary patterns were very similar across the two age groups. The six food items with the highest magnitude and the six food items with the lowest magnitude were chosen to reflect consumption of both more and less healthy foods; twelve-item scores were calculated using the corresponding coefficients and frequency of consumption of these foods. Agreement between the dietary quality scores for both pre-schoolers and primary school-aged children was considered good (Bland–Altman limits of agreement –1·05 to 1·05 sd and 1·10 to 1·10 sd, respectively). Correlations between dietary scores and biomarkers were in the expected direction for both age groups, with only minor attenuation observed for the 12-item scores compared with the 129-item scores. Higher prudent dietary scores were observed among primary school-aged girls, non-white children and children from more socio-economically advantaged backgrounds.

The resulting twelve discriminatory foods for pre-school and primary school-aged children have been used to derive the short FFQ in online Supplementary Figs. 1 and 2, respectively. Corresponding instructions on how to calculate the prudent diet score for each short FFQ are provided in online Supplementary Figs. 3 and 4. These abbreviated tools are built for ease of use to reduce participant burden and improve the quality and efficiency of dietary data collection in large research studies. Furthermore, the application of both FFQ does not require nutrient analysis and are therefore easily accessible to researchers without undertaking additional coding.

### Comparison with literature

The NDNS has previously been used to create an abbreviated FFQ for UK adolescents^(13)^ and one for UK adults^(47)^. The two 12-item FFQ produced in this study provide complementary tools to these previous measures and enable monitoring and dietary quality assessment among UK pre-school and primary schoolchildren. These tools demonstrate comparable methodological approaches (*a posteriori*) and dietary patterns to other child FFQ in the UK^(48,49)^ and in other countries^(50,51)^, including abbreviated FFQ used in Sweden^(23)^ and a multinational study^(24,52)^. Dietary patterns reported in these international studies show similarity to the prudent dietary patterns identified in this study, with an emphasis on fruits and vegetables and fibre-rich foods characterising a healthy dietary pattern and nutrient-poor, calorie-dense foods characterising less healthy dietary patterns^(23,24,48–51)^. Furthermore, the multinational study, which applied a twenty-three-item FFQ to assess dietary patterns among children across twelve countries, concluded that food groups within specified dietary patterns (i.e. ‘healthy or ‘less healthy’) were similar across differing nations^(24)^.

The *a posteriori* data-driven approach used to create these two 12-item FFQ was comparable to methods applied in prior studies^(13,23,24,48,50–52)^. This approach contrasts with *a priori*, or theory-driven, methods used frequently in the creation of dietary quality indices such as the well-known Healthy Eating Index^(53)^ or the Dietary Quality Index-International^(54)^. These indices can be limited by existing knowledge on food and nutrients, given that scores are based on adherence to established food models and tend to focus on single nutrients, which neglects the complexity of food combinations in meals ^(12,55)^.

Findings from this study showed higher prudent diet scores and better-quality diets among primary school-aged girls with a lower BMI, non-white ethnic groups and those living in more affluent contexts. These findings are consistent with our previous studies assessing sociodemographic relations to dietary patterns^(13,20)^. Similar associations have been observed in the UK, where NDNS data concluded girls aged 4–10 generally demonstrate a higher dietary quality compared with boys of the same age^(56)^. Moreover, children from households earning under £27 000 per year and living in more deprived areas exhibited lower quality dietary scores. This aligns with research in the UK and globally, indicating that socio-economic status is strongly linked to dietary quality, with children from lower socio-economic status backgrounds consuming fewer fruits and vegetables and higher amounts of refined sugar^(57,58)^. The alignment of our demographic findings and previous research supports the validity of the dietary scores developed in this paper.

The dietary quality scores derived from short FFQ for both age groups demonstrate similar agreement with biomarkers to the dietary score from 129 foods. Both positive and negative correlations between the comprehensive and abbreviated FFQ versions and biomarkers demonstrate the validity of the short FFQ to adequately assess dietary quality in relation to these nutrients. These associations align with expected health benefits as higher prudent dietary scores, typically indicative of increased fruit and vegetable consumption, are correlated with higher beta-carotene, total carotenoids and serum folate levels, with reductions in TAG and sodium levels. Increased levels of beta-carotene, total carotenoids and serum folate are commonly associated with decreased risk of disease^(34–38)^, while lower TAG and sodium levels have been linked to reduced onset of CVD^(38,39)^. Correlations seen in this study are similar to the previous short FFQ assessing dietary patterns in adolescence^(13)^, which further highlights the strength in this methodological approach to developing short, easy-to-use FFQ for population-level research.

### Strengths and limitations

A strength of this study lies in the use of a large, representative national dataset, the NDNS, to ensure representation of the UK population of pre-school and primary school-aged children. It is understood that self-reported dietary methods such as diet diaries are prone to inaccuracies, underreporting and possible biases^(59,60)^. However, best practice guidelines for dietary assessments have been observed throughout the NDNS to mitigate measurement errors as much as possible alongside the collection of additional variables (biomarkers and sociodemographic) to provide additional certainty of findings^(59,60)^.

While FFQ greatly reduce complexity in data collection and promote discovery of dietary patterns in epidemiological studies, they can be limited in their replicability^(14,61)^. Each culture and population will have access to different foods and dietary preferences. Children’s dietary patterns also evolve throughout childhood, and it should not be assumed that this methodology of dietary measurement will continue to remain relevant throughout childhood^(61)^. FFQ need to be formulated and validated for target populations, which is why different measures have been developed by this research team, alongside previously created adolescent and adult short FFQ, with consistency in validity demonstrated across all instruments. It is recommended to reconduct these measures every 5 years from the NDNS data to ensure relevance to current dietary intakes. An additional limitation of this includes the fact that the PCA analyses showed the most prominent patterns revealed only 3·0 and 3·4 % (pre-schoolers and primary schoolchildren, respectively) of variation within the data. However, a small proportion of variance explained would be anticipated when using a large number of variables, and these variance findings are aligned with previous studies^(13,62)^. Furthermore, PCA-derived FFQ require some subjective decisions including grouping of the dietary variables and naming of the resulting dietary patterns^(14)^; however, this coding was conducted by researchers with nutrition expertise. The small samples for the biomarker data used in this study (2–8 and 5–38 % for pre-school-aged and primary school-aged children, respectively) are a further limitation. The use of biomarker data, however, provided additional objective assurance regarding the validity of the PCA findings, particularly because expected correlations between nutritional biomarkers and prudent diet scores were observed. Lastly, while PPI engagement enabled feedback for both FFQ, the number of participants was limited and not fully representative of the target population. In future studies, the FFQ development would involve PPI with children and larger numbers.

### Interpretation and implications

Assessing dietary patterns in young children has become increasingly important in epidemiology research and public health practice to understand how dietary choices in early life may impact later life disease and health. Nutritional analyses have mostly focused on the relationship between a single nutrient or food and health outcomes but overlook associations and synergistic effects between nutrients and foods^(55)^. While research on individual nutrients is critical for certain clinical studies, assessing overall dietary patterns is pertinent for nutritional epidemiology studies where the determinants of non-communicable diseases are closely associated with various overlapping dietary influences^(63)^. These two 12-item FFQ capture the ability to determine dietary quality of UK pre-school and primary school-aged populations, lending themselves to ease of use in larger observational and intervention studies such as the ongoing ELL intervention currently active in Southampton primary schools. This is particularly crucial for informing policy and public health research, as these tools allow for continued monitoring of dietary quality among populations. This is a crucial need for assessment in population health research^(14,64)^.

The strong correlation between the 12-item prudent diet score and the 129-item score mirrors those in our previous studies of the creation of abbreviated FFQ for women and adolescents^(13,20)^, demonstrating the replicability of this methodology. Even with this reduced information, the twelve discriminatory foods can account for a large variation in diet as food consumption frequencies are correlated^(20)^. This association has also been confirmed in other studies analysing dietary patterns across the lifecourse^(13,20,65)^. Using the information in online Supplementary Figs. 1–4, researchers can examine consumption of both less and more healthy foods for group-level assessments. This flexibility allows for use within a variety of research situations including studies examining the reduction of unhealthy foods. Furthermore, eating patterns in early childhood can be predictive of eating patterns in early adulthood and beyond^(66)^. Therefore, these tools could be used to compare dietary scores collected in early years to dietary scores observed in adulthood. While the results of this study cannot be applied to other populations, the methodology used here could be used on nationally representative datasets in order to create alternative short FFQ.

Using these abbreviated FFQ will reduce the administrative burden of longer questionnaires, while increasing response rates and accuracy is anticipated from respondents due to the ease of completion^(23,49)^. This will be of particular importance with the ELL study, where these FFQ will be piloted with young children as self-completion tools to further understand child dietary patterns. Therefore, this approach is of benefit to both researchers and target populations, without compromising the integrity of dietary patterns analysis.

### Conclusion

This study successfully developed two concise 12-item FFQ: one for use among pre-school-aged children and a second for use among primary school-aged children. These measures are based upon the most distinctive foods associated with a prudent dietary pattern using a nationally representative dietary intake dataset for the UK population. Assessing dietary patterns of young children can be difficult; hence, the use of these short FFQ provides a clear advantage in terms of both data collection resource requirement and user-friendliness. The application of these measures can reduce the time and cost burden to both researchers and participants. Using the short FFQ in online Supplementary Figs. 1 and 2 and associated instructions in online Supplementary Figs. 3 and 4, prudent dietary scores can be calculated for UK pre-school and primary school-aged children. These measures can be used in a variety of research and public health practice settings.

## Supporting information

Mason et al. supplementary material 1 Mason et al. supplementary material

Mason et al. supplementary material 2 Mason et al. supplementary material

Mason et al. supplementary material 3 Mason et al. supplementary material

Mason et al. supplementary material 4 Mason et al. supplementary material

Mason et al. supplementary material 5 Mason et al. supplementary material

## Supplementary material

For supplementary material/s referred to in this article, please visit https://doi.org/10.1017/S0007114525103449.
