# Supplementary material for: Development of two short FFQ to assess diet quality in UK pre-school and primary school-aged children based on National Diet and Nutrition Survey data
Source: Br J Nutr. 2025 May 19;133(9):1287–96. doi: 10.1017/S0007114525103449 (PMC12229983; doi:10.1017/S0007114525103449)
Supplement: Mason et al. supplementary material 1 — Mason et al. supplementary material [file S0007114525103449sup001.docx]

**Supplementary Figure 1 12-item Food Frequency Questionnaire for UK Pre-schoolers**

We would like to know about some foods and how often they are eaten. Think about the foods eaten in the past **MONTH**. Please circle the number in one box in each row. If ‘more than once a day’ then specify number of times per day.

|  | **Over the past month how often have you eaten…** | **Never** | **Once a month** | **Once every two weeks** | **1-2 times per week** | **3-6 times per week** | **Once a day** | **More than once a day** | **Number of times per day** |
| --- | --- | --- | --- | --- | --- | --- | --- | --- | --- |
| 1. | **Salad and other raw vegetables**  *Cucumber, lettuce, raw peppers etc., not carrots* | 0 | 0.25 | 0.5 | 1.5 | 4.5 | 7 | 8 | 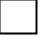 |
| 2. | **Uncooked tomatoes** | 0 | 0.25 | 0.5 | 1.5 | 4.5 | 7 | 8 | 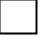 |
| 3. | **Apples and pears**  *Not tinned* | 0 | 0.25 | 0.5 | 1.5 | 4.5 | 7 | 8 | 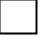 |
| 4. | **Peaches, plums, cherries, grapes and blueberries** | 0 | 0.25 | 0.5 | 1.5 | 4.5 | 7 | 8 | 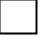 |
| 5. | **Crisps and savoury snacks**  *Crisps, popcorn (not sweet), twiglets, tortilla chips* | 0 | 0.25 | 0.5 | 1.5 | 4.5 | 7 | 8 | 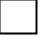 |
| 6. | **Burgers and kebabs not made at home**  *Not chicken* | 0 | 0.25 | 0.5 | 1.5 | 4.5 | 7 | 8 | 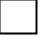 |
| 7. | **Crispy coated chicken or turkey**  *Fried chicken, chicken nuggets, turkey dinosaurs, chicken kievs, chicken burgers etc.* | 0 | 0.25 | 0.5 | 1.5 | 4.5 | 7 | 8 | 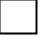 |
| 8. | **Chips**  *Purchased (such as frozen, oven or microwave) or takeaway/restaurant* | 0 | 0.25 | 0.5 | 1.5 | 4.5 | 7 | 8 | 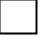 |
| 9. | **Other potato products not made at home**  *Waffles, croquettes, alphabites, fritters, hash browns, wedges, roast or sliced potatoes* | 0 | 0.25 | 0.5 | 1.5 | 4.5 | 7 | 8 | 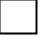 |
| 10. | **Tap water**  *Not squash* | 0 | 0.25 | 0.5 | 1.5 | 4.5 | 7 | 8 | 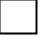 |
| 11. | **Fruit juice**  *100% single or mixed fruit juices, not squash or cordial* | 0 | 0.25 | 0.5 | 1.5 | 4.5 | 7 | 8 | 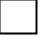 |
| 12. | **Soft drinks**  *Squash, cordial, fizzy drinks including diet versions, energy drinks not 100% fruit juice* | 0 | 0.25 | 0.5 | 1.5 | 4.5 | 7 | 8 | 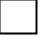 |
