# Supplementary material for: Development of two short FFQ to assess diet quality in UK pre-school and primary school-aged children based on National Diet and Nutrition Survey data
Source: Br J Nutr. 2025 May 19;133(9):1287–96. doi: 10.1017/S0007114525103449 (PMC12229983; doi:10.1017/S0007114525103449)
Supplement: Mason et al. supplementary material 3 — Mason et al. supplementary material [file S0007114525103449sup003.docx]

**Supplementary Figure 3 Instructions for creating 12-item prudent diet score** **for UK Pre-schoolers**

| **Food** | **Mean** | **SD** | **Coefficient** | |
| --- | --- | --- | --- | --- |
| Salad and other raw vegetables | 1.63 | 3.05 | 0.23 |  |
| Uncooked tomatoes | 0.64 | 1.50 | 0.20 |  |
| Apples and pears | 2.92 | 2.92 | 0.16 |  |
| Peaches, plums, cherries, grapes and blueberries | 2.36 | 3.05 | 0.20 |  |
| Crisps and savoury snacks | 1.98 | 2.24 | -0.09 |  |
| Burgers and kebabs not made at home | 0.18 | 0.62 | -0.09 |  |
| Crispy coated chicken or turkey | 0.81 | 1.34 | -0.17 |  |
| Chips | 1.11 | 1.44 | -0.18 |  |
| Other potato products not made at home | 0.60 | 1.33 | -0.11 |  |
| Tap water | 8.57 | 9.45 | 0.24 |  |
| Fruit juice | 3.00 | 5.12 | 0.18 |  |
| Soft drinks | 17.63 | 17.80 | -0.21 |  |

**Steps to derive 12-item prudent diet score**

- For each food variable in a new dataset, ensure the data are recorded as frequencies per week.
- Create ‘standardised’ food variables by subtracting the mean and dividing by the SD associated with that food in the above table.
- Create the 12-item prudent diet score by multiplying the coefficient for each food by the standardised food variables, and summing them, resulting in one score for each participant.
- This score has no units. Subtract the mean and divide by the SD of the score in the dataset to get a standardised score with internal standard deviation units.
- Alternatively, if the score is not normally distributed and a normally distributed score is required, consider transformations such as the logarithm (which may require addition of a constant) before internal standardisation as described in the bullet point above.
