# Supplementary material for: Development of two short FFQ to assess diet quality in UK pre-school and primary school-aged children based on National Diet and Nutrition Survey data
Source: Br J Nutr. 2025 May 19;133(9):1287–96. doi: 10.1017/S0007114525103449 (PMC12229983; doi:10.1017/S0007114525103449)
Supplement: Mason et al. supplementary material 4 — Mason et al. supplementary material [file S0007114525103449sup004.docx]

**Supplementary Figure 4 Instructions for creating 12-item prudent diet score** **for UK Primary School-Aged Children**

| **Food** | **Mean** | **SD** | **Coefficient** | |
| --- | --- | --- | --- | --- |
| Salad and other raw vegetables | 2.26 | 3.82 | 0.26 |  |
| Uncooked tomatoes | 0.72 | 1.56 | 0.23 |  |
| Uncooked carrots | 0.41 | 1.10 | 0.20 |  |
| Tropical fruit | 0.58 | 1.50 | 0.18 |  |
| Nuts and seeds | 0.37 | 1.59 | 0.19 |  |
| Crisps and savoury snacks | 2.99 | 2.82 | -0.11 |  |
| Crispy coated chicken or turkey | 0.99 | 1.67 | -0.15 |  |
| Meat pies and pastries not made at home | 0.56 | 1.56 | -0.09 |  |
| Chips | 1.67 | 1.82 | -0.16 |  |
| White bread | 5.84 | 4.38 | -0.10 |  |
| Tap water | 8.09 | 8.56 | 0.24 |  |
| Soft drinks | 15.64 | 14.04 | -0.19 |  |

**Steps to derive 12-item prudent diet score**

- For each food variable in a new dataset, ensure the data are recorded as frequencies per week.
- Create ‘standardised’ food variables by subtracting the mean and dividing by the SD associated with that food in the above table.
- Create the 12-item prudent diet score by multiplying the coefficient for each food by the standardised food variables, and summing them, resulting in one score for each participant.
- This score has no units. Subtract the mean and divide by the SD of the score in the dataset to get a standardised score with internal standard deviation units.
- Alternatively, if the score is not normally distributed and a normally distributed score is required, consider transformations such as the logarithm (which may require addition of a constant) before internal standardisation as described in the bullet point above.
