# Supplementary material for: Development of two short FFQ to assess diet quality in UK pre-school and primary school-aged children based on National Diet and Nutrition Survey data
Source: Br J Nutr. 2025 May 19;133(9):1287–96. doi: 10.1017/S0007114525103449 (PMC12229983; doi:10.1017/S0007114525103449)
Supplement: Mason et al. supplementary material 5 — Mason et al. supplementary material [file S0007114525103449sup005.docx]

**Supplementary Table 1 Principal component analysis coefficients all foods**

| **Food** | **Pre-schoolers** | **Primary school-aged** |
| --- | --- | --- |
|  | **(n = 1069)** | **(n = 2565)** |
| Pizza | 0.02 | 0.03 |
| Pasta (manufactured products and ready meals) | **-0.08** | -0.04 |
| Pasta (other, including homemade dishes) | **0.10** | **0.13** |
| Rice (manufactured products and ready meals) | 0.05 | 0.01 |
| Rice (other, including homemade dishes) | **0.13** | **0.13** |
| Other cereals | **0.10** | **0.07** |
| White bread (not high fibre or multiseed) | **-0.08** | **-0.10** |
| Wholemeal bread (3R) | **0.13** | **0.12** |
| Brown, granary and wheatgerm bread | **0.07** | 0.05 |
| Other bread | 0.05 | **0.09** |
| High fibre breakfast cereals | **0.11** | **0.10** |
| Other breakfast cereals | **-0.06** | **-0.07** |
| Biscuits (manufactured/retail) | **0.10** | **0.06** |
| Biscuits (homemade) | 0.04 | **0.08** |
| Fruit pies (manufactured) | 0.04 | -0.01 |
| Fruit pies (homemade) | 0.03 | 0.04 |
| Buns, cake and pastries (manufactured) | 0.05 | 0.05 |
| Buns, cake and pastries (homemade) | **0.11** | **0.09** |
| Cereal-based milk puddings (manufactured) | 0.01 | 0.02 |
| Cereal-based milk puddings (homemade) | 0.04 | 0.05 |
| Sponge puddings (manufactured) | -0.02 | 0.02 |
| Sponge puddings (homemade) | 0.04 | 0.01 |
| Other cereal-based puddings (manufactured) | 0.05 | 0.02 |
| Other cereal-based puddings (homemade) | **0.09** | **0.06** |
| Whole milk | **0.06** | 0.02 |
| Semi-skimmed milk | -0.04 | -0.04 |
| 1% milk | 0.01 | 0.00 |
| Skimmed milk | -0.01 | -0.01 |
| Infant formula | 0.05 | 0.02 |
| Cream (including imitation cream) | **0.06** | **0.09** |
| Other milk | 0.04 | **0.07** |
| Cottage cheese | **0.06** | 0.03 |
| Cheddar cheese | **0.08** | **0.11** |
| Other cheese | **0.12** | 0.05 |
| Yoghurt | **0.08** | **0.10** |
| Fromage frais and other dairy desserts (manufactured) | 0.05 | -0.01 |
| Dairy desserts (homemade) | 0.02 | 0.00 |
| Ice cream | 0.03 | 0.03 |
| Manufactured egg-products such as ready meals | 0.01 | 0.02 |
| Other eggs and egg dishes including homemade | **0.13** | **0.11** |
| Butter | **0.11** | **0.09** |
| Polyunsaturated margarine | ***** | 0.00 |
| Polyunsaturated oils | 0.04 | 0.05 |
| Polyunsaturated low fat spread | -0.04 | -0.03 |
| Low fat spread not polyunsaturated | 0.02 | 0.00 |
| Block margarine | -0.01 | 0.00 |
| Soft margarine not polyunsaturated | 0.01 | 0.00 |
| Other cooking fats and oils not polyunsaturated | **0.16** | **0.10** |
| Reduced fat spread (polyunsaturated) | -0.03 | 0.00 |
| Reduced fat spread (not polyunsaturated) | -0.05 | **-0.07** |
| Ready meals/meal centres based on bacon and ham | ***** | 0.02 |
| Other bacon and ham (including homemade dishes) | 0.00 | **-0.06** |
| Manufactured beef products (including ready meals) | -0.02 | -0.01 |
| Other beef and veal (including homemade recipe dishes) | **0.08** | **0.06** |
| Manufactured lamb products (including ready meals) | -0.02 | 0.03 |
| Other lamb (including homemade dishes) | **0.07** | **0.08** |
| Manufactured pork products (including ready meals) | 0.01 | -0.01 |
| Other pork (including homemade recipe dishes) | **0.07** | 0.03 |
| Manufactured coated chicken/turkey products | **-0.17** | **-0.15** |
| Manufactured chicken products (including ready meals) | -0.01 | -0.03 |
| Other chicken/turkey (including homemade recipe dishes) | **0.12** | **0.08** |
| Liver and dishes | 0.01 | 0.04 |
| Burgers and kebabs purchased | **-0.09** | **-0.08** |
| Ready meals based on sausages | 0.00 | -0.01 |
| Other sausages | -0.05 | -0.04 |
| Meat pies and pastries (manufactured) | **-0.07** | **-0.09** |
| Meat pies and pastries (homemade) | 0.01 | 0.02 |
| Other meat products (manufactured including ready meals) | 0.01 | -0.02 |
| Other meat (including homemade recipe dishes) | 0.02 | 0.02 |
| White fish coated or fried | -0.04 | 0.00 |
| Manufactured white fish products (including ready meals) | -0.01 | 0.02 |
| Other white fish (including homemade dishes) | **0.10** | 0.05 |
| Manufactured shellfish products (including ready meals) | -0.01 | 0.01 |
| Other shellfish (including homemade dishes) | 0.01 | **0.08** |
| Manufactured canned tuna products (including ready meals) | 0.01 | 0.03 |
| Other canned tuna (including homemade dishes) | 0.02 | 0.05 |
| Manufactured oily fish products (including ready meals) | 0.04 | **0.07** |
| Other oily fish (including homemade dishes) | **0.09** | **0.07** |
| Carrots (raw) | **0.15** | **0.20** |
| Salad and other raw vegetables | **0.23** | **0.26** |
| Tomatoes (raw) | **0.20** | **0.23** |
| Peas not raw | 0.01 | 0.05 |
| Green beans not raw | **0.09** | **0.10** |
| Baked beans | **-0.09** | **-0.06** |
| Leafy green vegetables not raw | **0.10** | **0.13** |
| Carrots not raw | **0.07** | **0.08** |
| Tomatoes not raw | **0.06** | 0.05 |
| Beans and pulses (including ready meals and homemade dishes) | **0.11** | **0.14** |
| Meat alternatives (including ready meals and homemade dishes) | 0.05 | **0.12** |
| Other manufactured vegetables products (including ready meals) | 0.00 | 0.02 |
| Other vegetables (including homemade dishes) | **0.13** | **0.17** |
| Chips, purchased including takeaway | **-0.18** | **-0.16** |
| Other manufactured potato products fried/baked | **-0.11** | **-0.07** |
| Other fried/roast potatoes (including homemade dishes) | -0.04 | 0.00 |
| Other potato products and dishes (manufactured) | 0.02 | 0.00 |
| Other potatoes (including homemade dishes) | 0.02 | 0.02 |
| Crisps and savoury snacks | **-0.09** | **-0.11** |
| Nuts and seeds | **0.15** | **0.19** |
| Apples and pears not canned | **0.16** | **0.16** |
| Citrus fruit not canned | **0.13** | **0.14** |
| Bananas | **0.14** | **0.10** |
| Canned fruit in juice | **0.07** | 0.05 |
| Canned fruit in syrup | 0.01 | -0.02 |
| Cooked fruit | **0.06** | 0.05 |
| Dried fruit | **0.16** | **0.17** |
| Peaches, plums, cherries, grapes and blueberries | **0.20** | **0.17** |
| Strawberries and raspberries | **0.15** | **0.16** |
| Tropical fruit | **0.13** | **0.18** |
| Other fruit | **0.16** | **0.15** |
| Sugar | **-0.06** | -0.04 |
| Preserves | **0.10** | **0.09** |
| Sweet spread fillings and icing | 0.00 | 0.01 |
| Sugar confectionery | 0.00 | 0.00 |
| Chocolate confectionery | -0.04 | -0.03 |
| Fruit juice | **0.18** | **0.13** |
| Smoothies | 0.01 | **0.07** |
| Soft drinks | **-0.21** | **-0.19** |
| Coffee (made up weight) | -0.01 | -0.01 |
| Tea (made up) | **-0.08** | **-0.08** |
| Herbal tea (made up) | 0.04 | 0.05 |
| Bottled water still or carbonated | **0.07** | 0.04 |
| Tap water only | **0.24** | **0.24** |
| Beverages dry weight | 0.01 | **0.06** |
| Soup (manufactured/retail) | **-0.07** | -0.01 |
| Soup (homemade) | **0.10** | **0.06** |
| Nutrition powders and drinks | 0.02 | 0.01 |
| Savoury sauces, pickles, gravies and condiments | **0.11** | **0.14** |
| Commercial toddler drinks | 0.01 | 0.00 |
| Commercial toddler foods | 0.05 | 0.05 |
| % variance explained | 3.4% | 3.0% |

*Not consumed by any pre-schoolers in the sample

Coefficients in bold greater than 0.05 in magnitude
